# *Schistosoma japonicum* Soluble Egg Antigen Protects Against Type 2 Diabetes in *Lep*r^*db*/*db*^ Mice by Enhancing Regulatory T Cells and Th2 Cytokines

**DOI:** 10.3389/fimmu.2019.01471

**Published:** 2019-06-26

**Authors:** Chun-lian Tang, Xiao-hong Yu, Yan Li, Rong-hui Zhang, Jun Xie, Zhi-ming Liu

**Affiliations:** ^1^Wuchang Hospital Affiliated to Wuhan University of Science and Technology, Wuhan, China; ^2^Medical College of Wuhan University of Science and Technology, Wuhan, China

**Keywords:** *Schistosoma japonicum* soluble egg antigen, type 2 diabetes, regulatory T cells, cytokines, *Lep*r^*db*/*db*^ mice

## Abstract

Type 2 diabetes is a metabolic disorder characterized by persistently elevated glucose levels. There is no effective treatment strategy for this condition, and it poses a massive economic burden globally. *Schistosoma* soluble egg antigen (SEA)-induced immunomodulatory mechanisms have been reported in the treatment of autoimmune disease. This study aimed to determine the ability of *Schistosoma japonicum* SEA to protect against type 2 diabetes in *Lep*r^*db*/*db*^ mice and understand the associated mechanisms. The mice were divided into four groups: C57BL/6 (the normal group), SEA (C57BL/6 mice treated with SEA), *Lep*r^*db*/*db*^, and SEA and *Lep*r^*db*/*db*^ co-treatment groups. The mice in the SEA and co-treatment groups were injected with 50 μg of SEA (twice a week for 6 weeks), and the same volume of PBS was used as control. Blood glucose, insulin, and HOMA-IR levels were measured in all mice, which were sacrificed 6 weeks after the last SEA administration. Flow cytometry was used to detect the percentages of regulatory T cells in splenocytes. ELISA was used to detect the levels of IFN-γ, IL-2, IL-4, and IL-5 in cell culture supernatants. Compared with the mice in the *Lep*r^*db*/*db*^ group, the mice in the SEA + *Lep*r^*db*/*db*^ group exhibited significantly reduced insulin resistance, as evidenced by the enhancement of wound healing. The frequency of spleen regulatory T cells increased significantly after SEA administration; meanwhile, the secretion of IL-4 and IL-5 in spleen cells was elevated. These results indicate that SEA can reduce insulin resistance and provide new targets for the treatment of type 2 diabetes. The potential mechanisms might be associated with increases in regulatory T cells and Th2 cytokines in *Lep*r^*db*/*db*^ mice, which warrants further investigation.

## Introduction

Diabetes is a metabolic disorder syndrome caused by the dysfunction of insulin secretion. The global healthcare expenditure on patients with diabetes was estimated to be USD 850 billion in 2017. The International Diabetes Federation reported that “There is one diabetic among every 11 adults in the world.” The incidence may increase to 693 million in 2045, with more than 90% of cases being of type 2 diabetes (T2D) (1). The main pathological feature of T2D is insulin resistance, which causes persistently elevated glucose levels. T2D can be treated with diet, exercise, and medication; however, the effect of these measures is not obvious, and those affected require long-term or even lifetime insulin use (2). The pathogenesis of T2D has not been clarified, and there is no effective preventive or curative measure. Therefore, there is an urgent need to develop new treatment methods. Beura et al. (3) noted that environmental factors affect the immune state of the body. Among Asian Indians, epidemiological studies have reported an inverse correlation between the incidence of lymphatic filariasis and T2D, as demonstrated by lower levels of the pro-inflammatory cytokines IL-6 and GM-CSF (4). It has also been reported that schistosomiasis is negatively correlated with the incidence of diabetes. Furthermore, the incidence of diabetes in a schistosomiasis-infected group (14.9%) was significantly lower than that in an uninfected group (25.4%) (5). Previous schistosome infection was also found to be significantly correlated with a lower prevalence of metabolic syndrome and its components, including central obesity, hypertriglyceridemia, and low high-density lipoprotein cholesterol (6). Based on these findings, some helminths such as *Schistosoma* might be promising in the treatment of T2D by immunoregulation.

In recent years, *Schistosoma* infection and its by-products have received increased attention in the possible treatment of T2D. Hussaarts et al. (7) reported that in mice with chronic obesity induced by a high-fat diet, chronic infection with *Schistosoma mansoni* decreased body weight, fat aggregation, and adipocyte volume and improved adipose tissue sensitivity to insulin in peripheral tissues. Luo et al. (8) also reported that chronic *S. japonicum* infection with praziquantel chemotherapy protected against metabolic syndrome via a mechanism involving the enhancement of the Th2-type immune response. Hams et al. (9) reported that ω-1, derived from recombinant *S. mansoni* eggs, improved the metabolic status of obese mice by the release of the Th2-type cytokine IL-33. *S. japonicum* soluble egg antigen (SEA) is an antigen secreted by the eggs, and it can exude through the eggshell to activate sensitized T cells of the immune system. In this study, we used *Lep*r^*db*/*db*^ mice to study the effect and mechanism of *S. japonicum* SEA on T2D. The results demonstrated that SEA can reduce insulin resistance in *Lep*r^*db*/*db*^ mice, which may be correlated with the enhancement of regulatory T cells (Tregs) and Th2 cytokines.

## Materials and Methods

### Animals and Parasites

Male C57BL/6 mice and *Lep*r^*db*/*db*^ mice (C57BL/6 mice with the diabetes *db* mutation in the leptin receptor) (aged 6 weeks) were obtained from the Model Animal Research Center of Nanjing University and kept in a specific pathogen-free environment. *Lep*r^*db*/*db*^ mice are overweight, have severe insulin resistance, exhibit elevated liver enzyme levels, and serve as a model for T2D (10). The mice were divided into four groups: C57BL/6 (the normal group), SEA (C57BL/6 mice treated with SEA), *Lep*r^*db*/*db*^, and SEA and *Lep*r^*db*/*db*^ co-treatment groups. The experiment was performed in triplicate (*n* = 6 mice per group) and was approved by the Committee on Animal Research of Wuchang Hospital (No. 2018-0032). Snails of the Chinese strain of *S. japonicum*-infected *Oncomelania hupensis* were bought from the JiangShu Institute of Parasitic Diseases (Wuxi, China). Cercariae were collected from infected snails, and the preparation of SEA was based on a previous publication (11). Briefly, for SEA, eggs were obtained from the livers of infected mice that were homogenized and washed with phosphate-buffered saline on ice. Polymyxin B agarose beads (Sigma-Aldrich, St. Louis, MO, USA) were used for sterile filtration and endotoxin removal to <1 EU/mg.

### Immunization Schedule and Metabolic Measurements

The 6-weeks-old mice in the SEA group and co-treatment group were intraperitoneally injected with 50 μg of SEA (twice a week for 6 weeks), and the same volume of PBS was used as a control. Mice were killed 6 weeks after SEA administration, and the blood glucose and blood fasting insulin concentrations were measured after a 12–16 h overnight fast. Blood glucose levels were measured using an automatic glucose monitor, and the serum insulin concentration was measured with a mouse insulin ELISA kit (Shibayagi Co., Ltd., Shibukawa, Japan). Insulin resistance refers to a phenomenon wherein the body's normal response to insulin is hampered, that is, insulin sensitivity decreased. We detected insulin resistance by using homeostasis model assessment as an index for insulin resistance (HOMA-IR), which is a method used to calculate insulin resistance according to the following formula: fasting insulin (μU/L) × fasting glucose (nmol/L)/22.5.

### Flow Cytometric Analysis of Tregs in Splenocytes

To measure the percentage of Tregs, a single-cell suspension of splenic cells was prepared according to a previously described method (12). The cells were stained with a mouse Treg staining kit (eBioscience) and analyzed with Cell Quest software. Cell suspensions were stained by adding 1 μL of FITC-labeled anti-mouse CD4, 1 μL of PE-labeled anti-CD25 mAb, and 2 μL of APC-labeled anti-mouse Foxp3 (clone PC61), and APC-conjugated rat IgG2α served as isotype control. Then sorted for the three target populations by flow cytometry using a FACSCalibur system (Becton Dickinson).

### Cytokine Measurement

Spleens were removed from mice 6 weeks after the last SEA administration, and 5 ×10^6^ cells/well were cultured for 72 h at 37°C in 5% CO_2_ in the presence of 5 μg/mL SEA. The supernatants were then collected, and the IFN-γ, IL-2, IL-4, and IL-5 cytokines were measured with ELISA kits (eBioscience) according to the manufacturer's instructions.

### Statistical Analysis

All data are expressed as the mean ± SD and were analyzed with SPSS 17.0. ANOVA was used for comparisons among different groups. A value of *P* < 0.05 was considered significant.

## Results

### Effect of SEA on T2D in *Lepr^*db*/*db*^* Mice

**Figure 1:**
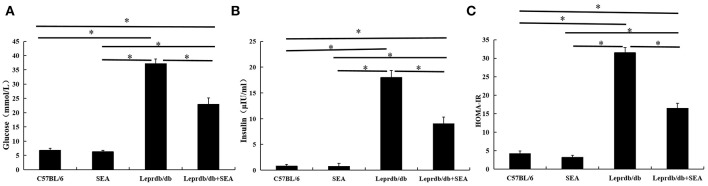
Improvement in insulin sensitivity after SEA administration in *Lep*r^*db*/*db*^ mice. **(A)** blood glucose; **(B)** serum insulin; **(C)** HOMA-IR. The mice were divided into four groups: C57BL/6 (normal group), SEA (C57BL/6 mice treated with SEA), *Lep*r^*db*/*db*^, and SEA + *Lep*r^*db*/*db*^ co-treatment groups. The experiment was performed in triplicate (*n* = 6 mice per group). ^*^denotes *P* < 0.05.

Among mutant mice, blood glucose (Figure 1A) and serum insulin (Figure 1B) levels in the *Lep*r^*db*/*db*^ group treated with SEA were significantly lower than those in untreated *Lep*r^*db*/*db*^ mice. Accordingly, HOMA-IR indicators also improved (Figure 1C). These results indicate that SEA improved insulin sensitivity in *Lep*r^*db*/*db*^ mice.

**Figure 2:**
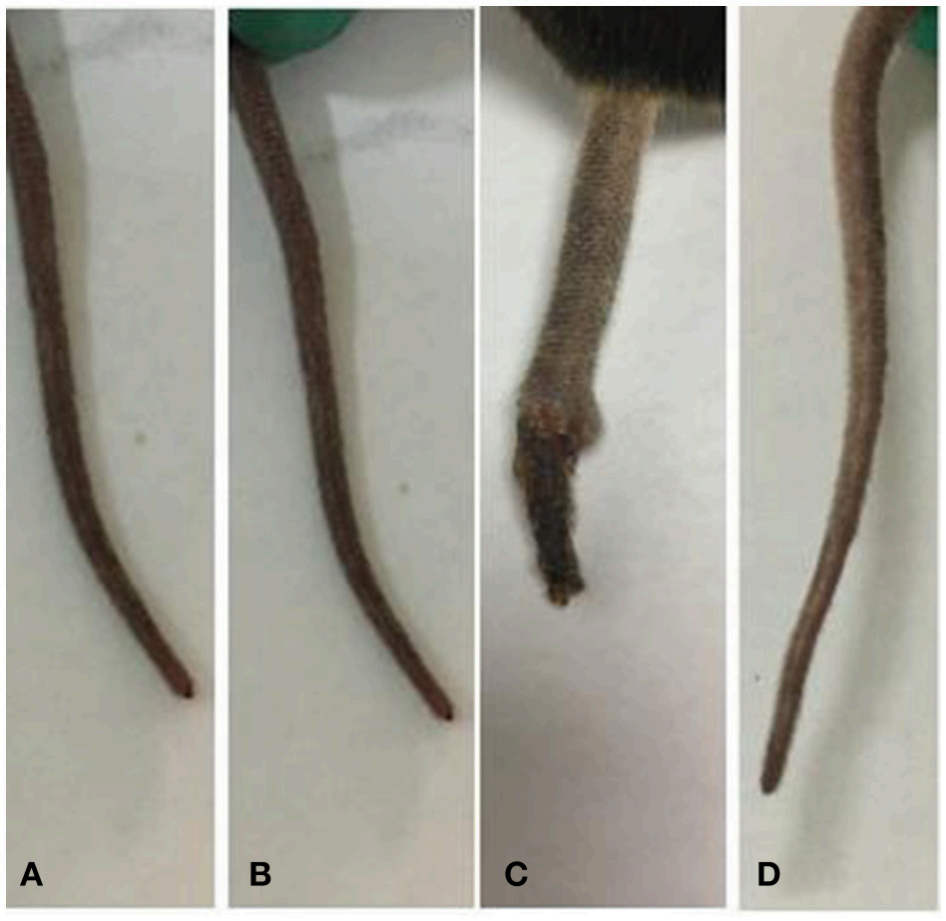
Tail wounds readily healed after SEA treatment. **(A)** C57BL/6 group; **(B)** SEA group; **(C)** *Lep*r^*db*/*db*^ group; **(D)** *Lep*r^*db*/*db*^ + SEA group.

Blood glucose levels were detected weekly using blood extracted from the caudal vein. This procedure caused a wound on the tail of the mice. The tail wounds of *Lep*r^*db*/*db*^ mice healed slowly, while those of the other three groups healed within a week. Wound is also an obvious complication of T2D, and the treatment of such complications is important in the treatment of T2D. As shown in Figure 2, compared with that in untreated *Lep*r^*db*/*db*^ mice, wound healing in the SEA-treated *Lep*r^*db*/*db*^ mice was markedly better, indicating that SEA markedly improved the complications of T2D such as wound healing.

### Effect of SEA on the Frequency of Tregs in Spleen Cells

**Figure 3:**
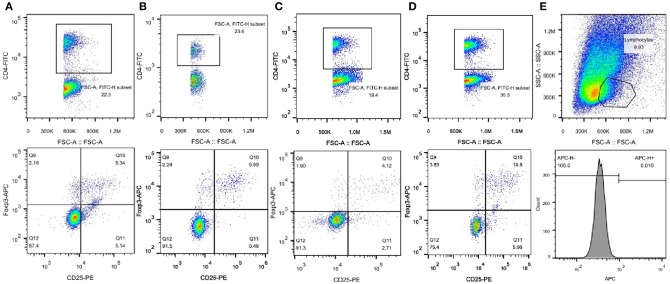
Representative FACS results of Tregs from one experiment. **(A)** C57BL/6 group; **(B)** SEA group; **(C)** *Lep*r^*db*/*db*^ group; **(D)** *Lep*r^*db*/*db*^+ SEA group. Upper panels, the numbers denote the frequency of CD4^+^ T cells in splenocytes. Lower panels, the right upper quadrant indicates the frequency of CD25^+^Foxp3^+^ T cells from CD4^+^ lymphocytes. **(E)** Upper panel, the numbers denote the frequency of lymphocytes. Lower panel, APC-conjugated rat IgG2α served as isotype control.

**Figure 4:**
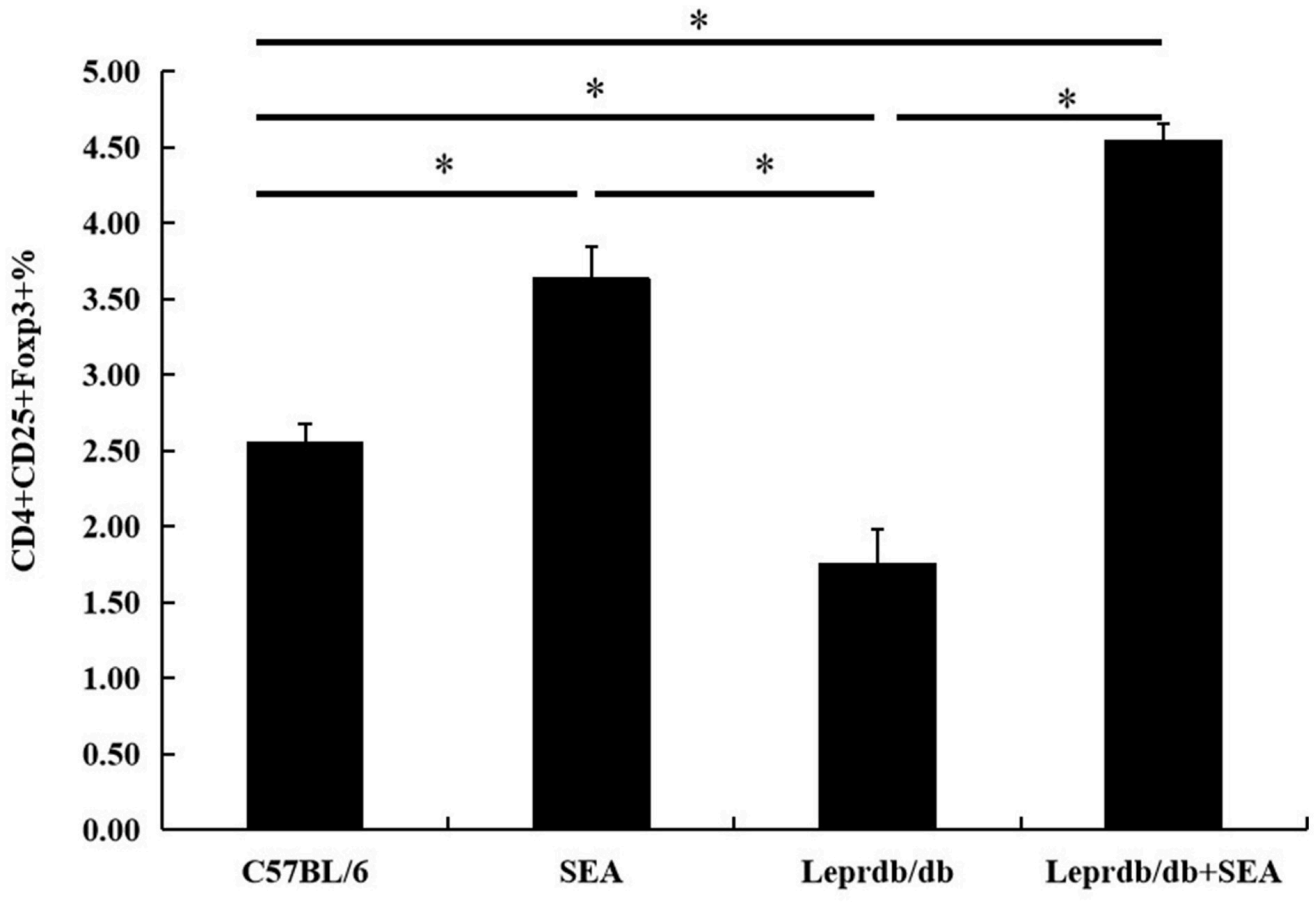
Effect of SEA on the frequencies of Tregs within total splenocytes. All data are presented as the mean ± SD. The experiment was performed three times (*n* = 6 mice per group). ^*^denotes *P* < 0.05.

As shown in Figures 3, 4, compared with that in C57BL/6 mice, the frequency of Tregs was significantly lower in the *Lep*r^*db*/*db*^ group and significantly higher in the SEA and co-treatment groups (*P* < 0.05). The frequency of Tregs in the SEA and co-treatment groups was also significantly higher than that in the *Lep*r^*db*/*db*^ group mice (*P* < 0.05).

### Cytokine Production by Splenocytes After SEA Administration

**Figure 5:**
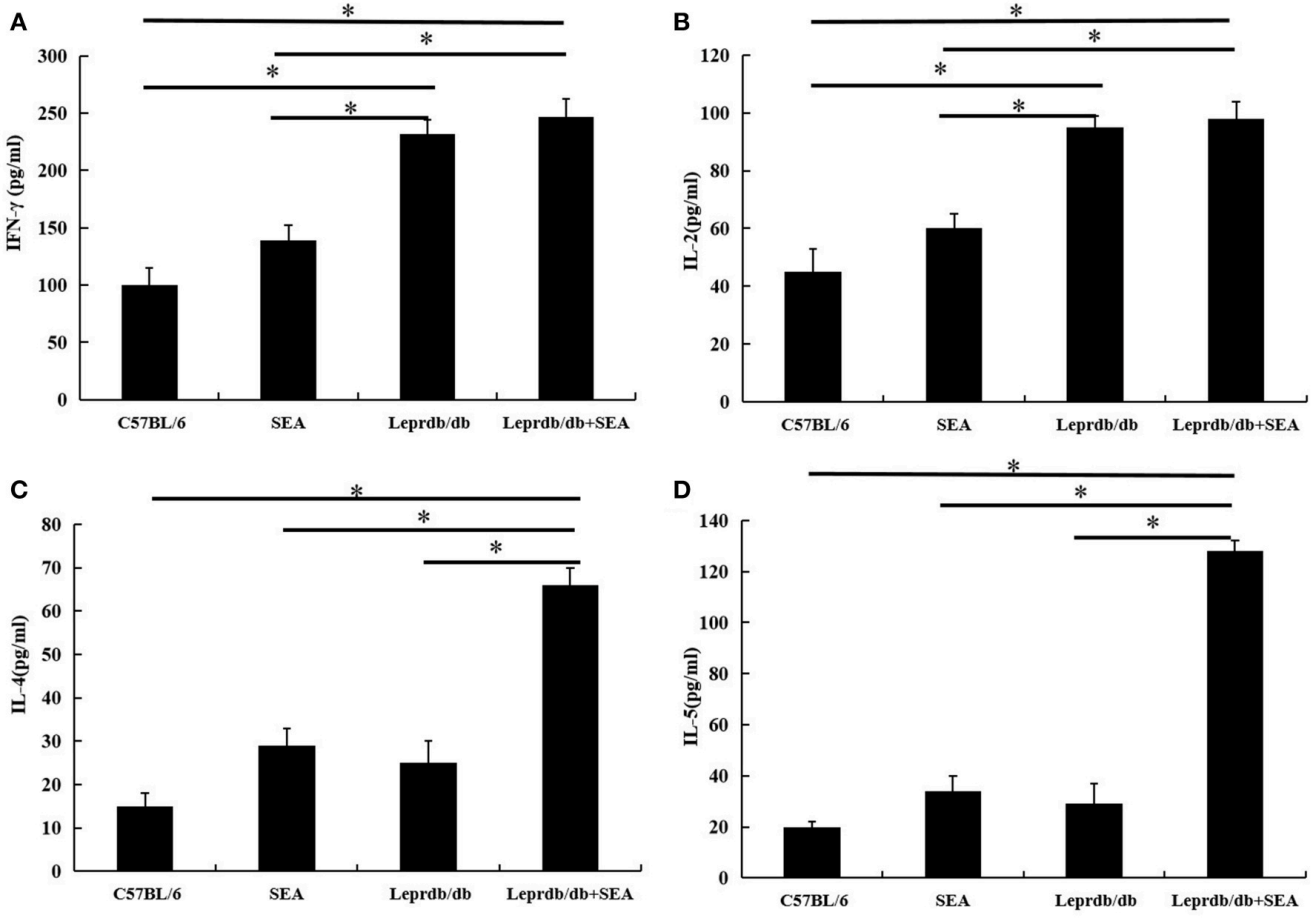
The expression levels of the cytokines IFN-γ **(A)**, IL-2 **(B)**, IL-4 **(C)**, and IL-5 **(D)** were determined by ELISA. Data are presented as the mean ± SD from triplicate experiments. ^*^denotes *P* < 0.05.

As shown in Figure 5, the levels of the Th1 cytokines IFN-γ and IL-2 in the *Lep*r^*db*/*db*^ group were significantly higher than those in the C57BL/6 group (*P* < 0.05), but there was no significant difference in the levels of Th2-type cytokines. Moreover, the levels of the Th2 cytokines IL-4 and IL-5 in the *Lep*r^*db*/*db*^+ SEA group were significantly higher than those in the *Lep*r^*db*/*db*^ group (*P* < 0.05). However, there was no significant difference in the levels of Th1-type cytokines.

## Discussion

Although *Schistosoma* was found to be beneficial to T2D in this study, it is important to note that *Schistosoma* spp. can cause many diseases. Therefore, *Schistosoma*-based approaches might not be readily accepted psychologically by the patient (13). Nevertheless, the development of schistosomiasis derivatives has opened up possibilities for a new treatment strategy for T2D (14). Studies on SEA-induced immunomodulatory mechanisms may contribute to the development of new methods for the treatment of inflammatory diseases such as inflammatory bowel disease, non-obese diabetes (NOD), and collagen-induced arthritis. It has been reported that SEA-treated DC exosomes attenuate the severity of acute DSS-induced colitis in mice more effectively than DC exosomes do (15). In NOD mice, *S. mansoni* SEA has been shown to prevent diabetes-induced changes in APCs and enhance Th2 and Treg responses (16). SJMHE1, an immunomodulatory peptide of *S. japonicum*, has also been reported to suppress the clinical signs of collagen-induced arthritis in mice and to block joint erosion progression by decreasing IFN-γ and TNF-α and increasing IL-10, TGF-β, and Treg levels (17). SEA is an antigen secreted by the eggs of *S. japonicum*, and it represents an admixture of many different proteins, including IPSE/alpha-1 and omega-1 (18). The start time of SEA administration and the duration of immunization have obvious effects on the treatment. Zaccone et al. (19) reported that continuous immunization four times and early use at 4 weeks old can be effective in NOD mice. Based on the literature (7–9, 19), SEA was administered at a dose of 50 μg twice a week for 6 weeks in this study. The results of this study demonstrated that compared with that in *Lep*r^*db*/*db*^ mice, the blood sugar level in the co-treatment group decreased significantly. Thus, SEA can lower insulin resistance in *Lep*r^*db*/*db*^ mice, which is a basic pathological characteristic of T2D. The results of this study also indicated that compared with those in the *Lep*r^*db*/*db*^ group, the tail wounds in the *Lep*r^*db*/*db*^+ SEA group markedly healed. SEA plays a key role in hepatic fibrosis by inducing TGF-β, which helps in wound healing (20). Thus, these results indicate that SEA has certain therapeutic effects on the complications of T2D, such as insulin resistance and wound healing.

However, we acknowledge an important limitation of this study regarding the choice of the mouse model, *Lep*r^*db*/*db*^ mice, which develop congenital diabetes because of a gene deletion. The blood sugar level in *Lep*r^*db*/*db*^ mice is very high, and after SEA treatment, blood sugar levels decreased significantly. However, the levels were still significantly higher than those in the normal group. Thus, perhaps it was not the best model for this study; therefore, streptozotocin and high-sugar and high-fat-induced T2D models can be used for further investigations.

To further study the mechanism of the effects of SEA on T2D, we used flow cytometry to measure the percentage of Tregs in splenic lymphocytes. Sakaguchi et al. (21) identified Tregs for the first time as a subset of CD4^+^ T cells that express IL-2Ra (CD25). Because Foxp3 is a specific marker of Tregs, we used the percentages of CD4^+^CD25^+^Foxp3^+^ T cells to evaluate Tregs in the spleens of all mouse groups (22). The result demonstrated that the frequency of Tregs in the *Lep*r^*db*/*db*^ group was significantly lower than that in C57BL/6 mice but was higher after SEA administration. Tregs have important immunosuppressive functions through the cytokines IL-10 and TGF-β. Gao et al. (23) reported that maintaining a higher level of IL-10 through gene transfer could be an effective strategy in preventing diet-induced obesity. Moreover, Tregs expressing the TGF-β-dependent latency-associated peptide reduce insulin resistance in leptin-deficient ob/ob mice (24). There is, in fact, a close relationship among SEA, Tregs, and T2D. In a previous study, compared with that in infected control groups, the percentage of Tregs in the group that received multiple doses of SEA immunization increased significantly 8 and 16 weeks post infection (25). SEA-induced B10 cells promote Treg amplification and induce IL-4 secretion but inhibit IL-17 production (26). Tregs are abundant in the lean adipose tissue of mice, but their number was significantly lower in the insulin resistance animal model due to decreased CCR1, CCR2, and CXCR6 expression, which might be responsible for the Treg-specific accumulation. The difference in Tregs between lean and obese models indicated that Tregs may have a therapeutic effect on T2D (27). Interestingly, insulin resistance is associated with a sharp decrease in Treg cells in several animal models of obesity such as leptin-deficient mice (*Lep*r^*ob*/*ob*^), mice heterozygous for the yellow spontaneous mutation, and male mice chronically fed a high-fat diet (HFD) (24). This study had similar results. *In vivo*, IL-2/anti-IL-2 complexes can improve insulin sensitivity in obese mice by promoting the expansion of Tregs (28). A treatment that specifically increases Tregs may be useful for the treatment of insulin resistance (29), and pioglitazone, a drug used to treat T2D, can increase insulin sensitivity by stimulating PPAR-γ signaling in Tregs, resulting in an increased frequency of Tregs in adipose tissue (30). Therefore, in this study, SEA may have reduced insulin resistance by inducing Tregs. Zaccone et al. (31) reported that blocking Tregs in splenocytes from SEA-treated donors restored the ability to transfer diabetes. SEA was shown to inhibit type 1 diabetes in NOD mice by inducing Tregs, which increased the expression of integrin beta8, TGF-β, and galectins. SEA was shown to prevent diabetes in NOD mice by upregulating bioactive TGF-β on T cells with the subsequent proliferation of Tregs (16).

Leptin, a pro-inflammatory adipokine, can increase and inhibit the production of circulating Th1-type and Th2-type cytokines, respectively (32). Th2 helper T cells secrete the cytokines IL-4, IL-5, IL-9, IL-10, and IL-13. The levels of Th2 in both adipose tissue and peripheral blood were reported to be negatively correlated with systemic insulin resistance (33). IL-4 was used to treat diet-induced obese mice and protected them from weight gain and glucose intolerance by activating the STAT6 pathway (34). Analyses of the immune response induced by injecting SEA into mice revealed Th2 responses (35). SEA has been shown to promote a strong Th2 response *in vitro* (36) and *in vivo* (37). In addition, ω-1 allows the ribosome and messenger RNA to skew the immune response toward a Th2 distribution (38). Lacto-N-fucopentaose III from SEA was also shown to induce a type 2 immune response (39). In another study, ELISA results demonstrated that there was a significant increase in the Th2 immune response, but there were no significant differences in Th1 immunoreactivity; the host mounted a Th1 response early in infection before shifting to a Th2 response 4 weeks later (40). In this study, we demonstrated that SEA administration increased the Th2-type immune response but not the Th1-type immune response. Th1 cytokine is positively correlated with markers of obesity and glucose tolerance in T2D patients (41). Therefore, in this study, SEA may have reduced insulin resistance by inducing Th2 responses or decreasing the Th1/Th2 ratio. In addition, the mechanism underlying the effects of SEA on T2D may be related to the innate immune system. SEA activates M2 macrophages via the STAT6 and PI3K pathways (42) and infiltration of eosinophils in adipose tissue (9), which plays an important role in maintaining insulin sensitivity in SEA.

In conclusion, soluble antigens of *S. japonicum* eggs can treat T2D by enhancing Tregs and Th2-type immune responses. Meanwhile, T2D is associated with many complications such as diabetic foot disease, and SEA administration may improve healing (43). SEA can be added to the treatment strategy for such patients and may have additional practical benefits.

## Ethics Statement

The experiment was approved by the Committee on Animal Research of Wuchang Hospital (No. 2018-0032).

## Author Contributions

CT and XY were responsible for the project planning and data analysis. YL and RZ were responsible for carrying out the experiments. JX and ZL were mainly responsible for the literature search and paper writing.

### Conflict of Interest Statement

The authors declare that the research was conducted in the absence of any commercial or financial relationships that could be construed as a potential conflict of interest.
